# Trends and Frontiers of Research on Cancer Gene Therapy From 2016 to 2020: A Bibliometric Analysis

**DOI:** 10.3389/fmed.2021.740710

**Published:** 2021-10-26

**Authors:** Shoushan Hu, Alifu Alimire, Yancheng Lai, Haonan Hu, Zhuo Chen, Yi Li

**Affiliations:** State Key Laboratory of Oral Diseases and National Clinical Research Center for Oral Diseases, Department of Head and Neck Oncology, West China Hospital of Stomatology, Sichuan University, Chengdu, China

**Keywords:** bibliometrics, CiteSpace, cancer gene therapy, gene delivery, ovarian cancer

## Abstract

**Background:** With rapid development in molecular biology techniques and a greater understanding of cancer pathogenesis, the growing attention has been concentrated on cancer gene therapy, with numerous articles on this topic published in recent 5 years. However, there is lacking a bibliometric analysis of research on cancer gene therapy. Therefore, the aim of the present study was to conduct a bibliometric analysis to provide the trends and frontiers of research on cancer gene therapy during 2016–2020.

**Methods:** We utilized CiteSpace 5.7.R5 software to conduct a bibliometric analysis of publications on cancer gene therapy published during 2016–2020. The bibliometric records were obtained from the Web of Science Core Collection.

**Results:** A total of 4,392 papers were included in the bibliometric analysis. Materials Science and Nanoscience and Nanotechnology took an increasing part in the field of cancer gene therapy. Additionally, WANG W was the most productive author, while ZHANG Y ranked top in terms of citations. Harvard Medical School and Sichuan University ranked top in the active institutions. *P NATL ACAD SCI USA* was identified as the core journal in the field of cancer gene therapy. “Ovarian cancer” was found to be the latest keyword with the strongest burst. The keyword analysis suggested that the top three latest clusters were labeled “gene delivery,” “drug delivery,” and “gene therapy.” In the reference analysis, cluster#2 labeled “gene delivery” held a dominant place considering both the node volume and mean year.

**Conclusion:** The academic attention on cancer gene therapy was growing at a dramatically high speed. Materials Science and Nanoscience and Nanotechnology might become promising impetus for the development of this field. “Gene delivery” was thought to best reflect the research frontier on cancer gene therapy. The top-cited articles on gene delivery were focused on several novel non-viral vectors due to their specialty compared with viral vectors. “Ovarian cancer” was likely to be the potential research direction. These findings would help medical workers conduct further investigations on cancer gene therapy.

## Introduction

With the aging of the global population, the overall incidence of new cancer cases will continue to increase (1, 2). As a traditional cancer therapy, surgical excision followed by chemoradiotherapy can improve the overall survival rate, but long-term chemotherapy will increase pain and side effects (3, 4). The current situation highlights the need for a new treatment strategy for cancer (5).

Cancer gene therapy is defined as an approach to treat cancer by transferring therapeutic nucleic acids to tumor cells or correcting defective genes (6). Since 1990 when the first clinical research of gene transfer technology was reported, revolutionary technological progress has taken place in cancer gene therapy (7). So far, cancer gene therapy has extended to many branches, including tumor suppressor gene therapy (8), gene silencing therapy (9), suicide gene therapy (10), immune gene therapy (11), anti-tumor angiogenesis therapy (12), oncolytic therapy (13), etc. It was reported that some gene therapy drugs have been applied in clinical practice for the treatment of various cancers (8, 14), such as head and neck tumor (15), pancreatic cancer (16), liver cancer (17), etc.

Bibliometrics is a quantitative analysis of publications and has been employed in a wide range of literature (18–21). Bibliometrics can help researchers to track the trends and frontiers in a certain field by identifying the high-impact articles, journals, research groups and institutions that make predominant contributions (22). With the rapid development in molecular biology techniques and a greater understanding of cancer pathogenesis, the growing attention has been concentrated on cancer gene therapy, with a number of papers on this topic published in recent 5 years. Whereas, so far, none has conducted a bibliometric analysis on cancer gene therapy. In order to make up for this deficiency, we searched the studies on cancer gene therapy published during 2016–2020 and conducted a bibliometric analysis to identify the trends and frontiers in this field.

## Materials and Methods

**Figure 1:**
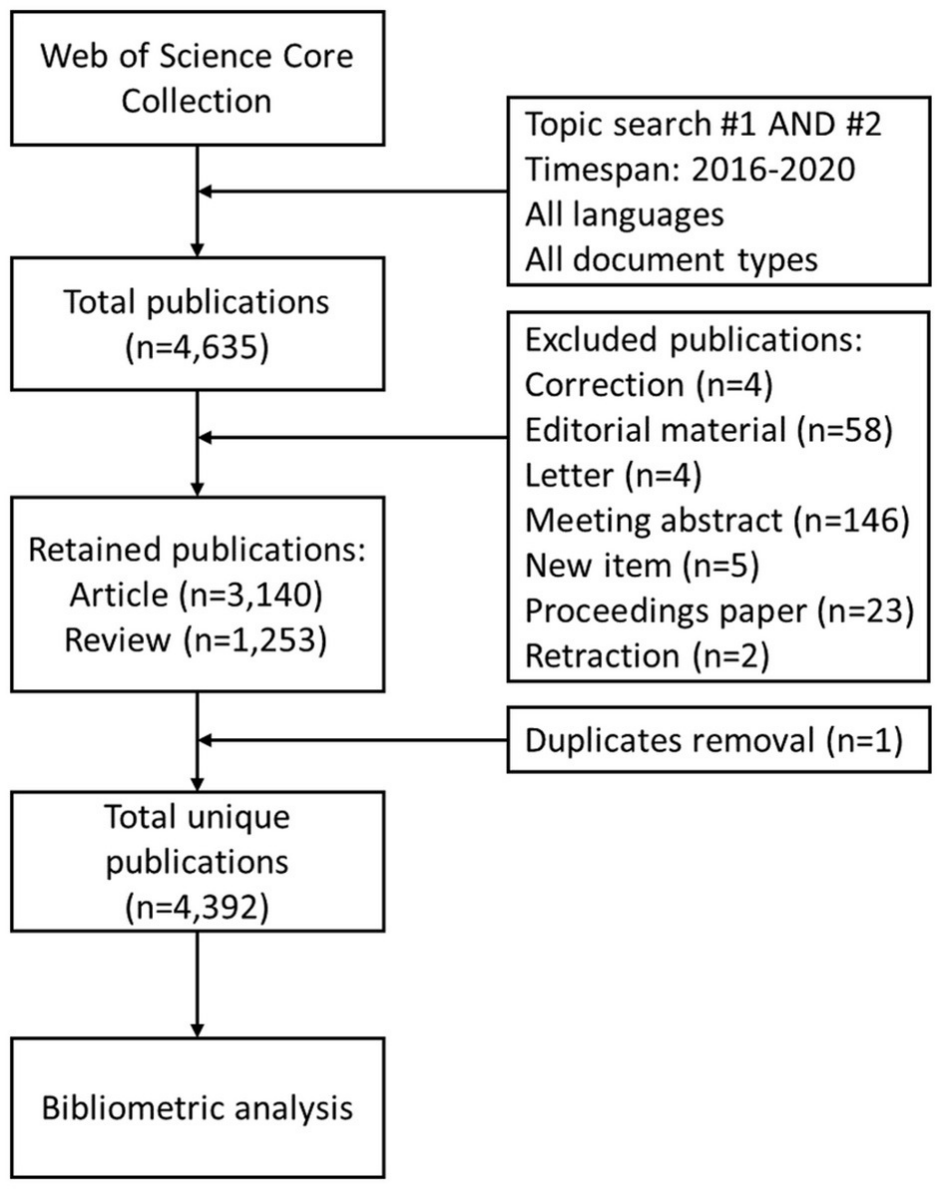
The flowchart illustrating the search strategy and selection process in this study. Topic search #1 = (“Genetic Therapy” OR “Genetic Therapies” OR “DNA Therapy” OR “DNA Therapies” OR “Gene Therapy” OR “Gene Therapies” OR “RNAi-Based Therapeutics” OR “RNAi-Based Therapy” OR “RNAi Based Therapy” OR “RNAi-Based Therapies” OR “RNAi Therapy” OR “RNAi Therapies”); Topic search #2 = (Neoplasia OR Neoplasias OR Neoplasm OR Neoplasms OR Tumors OR Tumor OR Cancer OR Cancers OR Malignancy OR Malignancies OR Carcinoma OR Carcinomas).

Publications on cancer gene therapy during 2016–2020 were identified from the Web of Science Core Collection. The search strategy and the inclusion criteria were detailed in Figure 1. The entire process of the data search and selection was conducted independently by two authors, and any discrepancy was resolved through consultation with the third author.

Bibliographic information of every included publication was collected, including the year of publication, the number of total citations, subject category, author, journal, institution, country of origin, and keywords. CiteSpace 5.7.R5 (Drexel University, Philadelphia, USA) is a useful tool for bibliometric analysis, which serves to visualize the bibliographic information. The numbers of annual citations and publications were obtained from the Web of Science Core Collection, which were then illustrated in a line chart to show the publication trends. In addition to the number of publications or/and citations, the centrality index is utilized to evaluate the impact of a(an) discipline, journal, author, institution, or country (23). In the network maps, the node with high centrality suggested that it was highly connected to other nodes or connected between two different groups of nodes (20). The purple outer ring of the node reflected that the centrality of the node was more than 0.1, indicating its prominent influence (21, 24). Additionally, to increase the readability of the information, the network maps of keywords and journals were trimmed by the pathfinder algorithm (25).

The results of the bibliometric analysis were shown in form of knowledge network maps. In the network maps, each node represents an item, such as a keyword, author, country, institution, journal or reference, and the frequency of occurrence was reflected by the node size. Each link between two nodes represents the co-occurrence relationship between two items, and the thicker line reflects more co-occurrence frequency between the two nodes. The nodes with a purple ring were thought to be the turning points in the network map, indicating the high impact of the items represented by the nodes.

## Results

### Analysis of Publication Trends and Subject Categories

**Figure 2:**
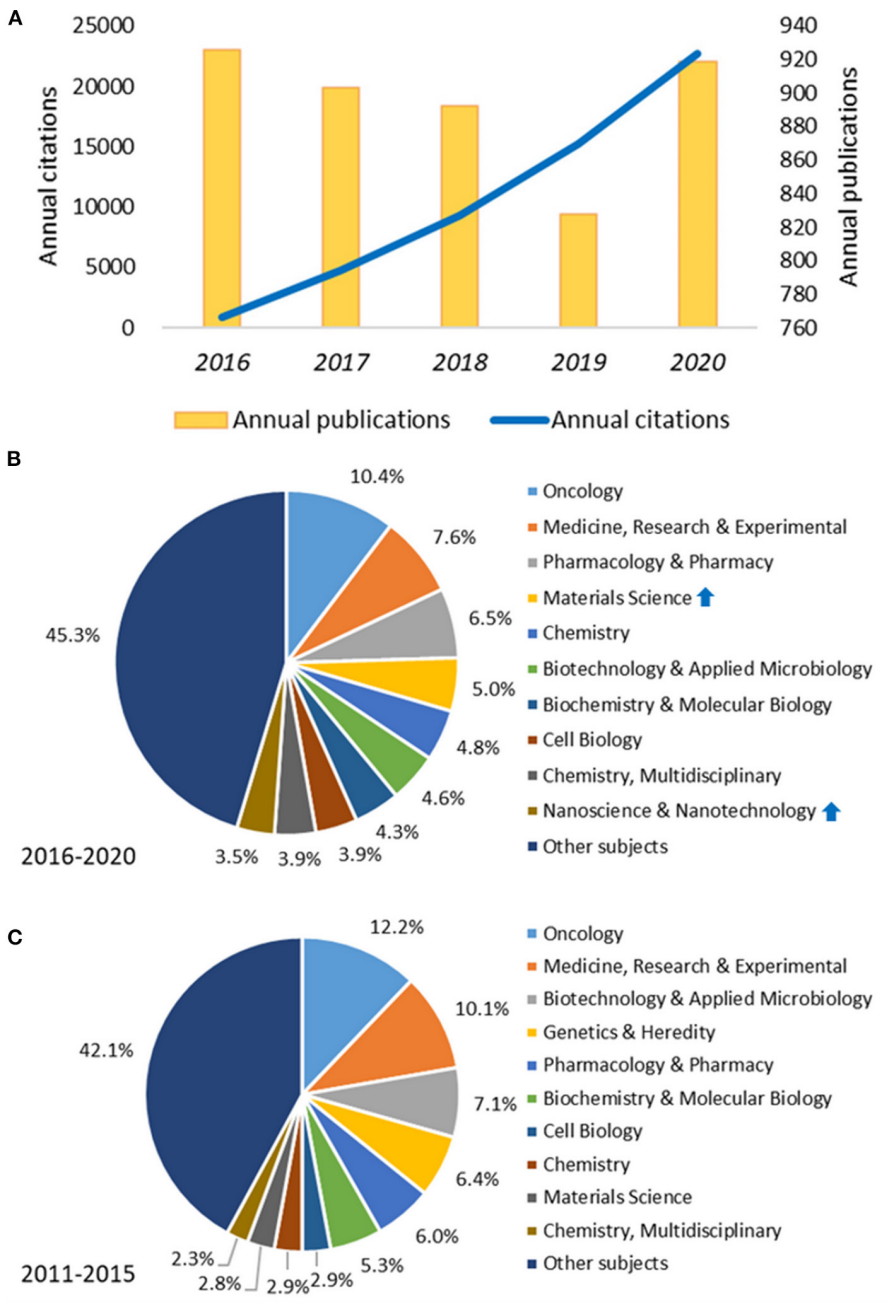
Analysis of publication trend and distribution changes of subject categories. **(A)** Trends in the number of publications and citations on cancer gene therapy from 2016 to 2020; **(B)** Distribution characteristics of subject categories on cancer gene therapy from 2016 to 2020; **(C)** Distribution characteristics of subject categories on cancer gene therapy from 2011 to 2015. The blue arrows denoted a remarkable increase in the proportion of subjects “Materials Science” and “Nanoscience and Nanotechnology” during 2016–2020 compared to that during 2011–2015.

The annual distribution of publications shows a significant decline in the number of publications from 2016 to 2019 (Figure 2A). Especially, in 2019, the number of publications was 828, a decrease of 10.58% compared to 2016 (*n* = 926). In 2020, there was a rebound in the number of publications, reaching 919. Despite the yearly decline in the number of annual publications from 2016 to 2019, the number of literature citations increased each year, rising from 868 citations in 2016 to 15,267 citations in 2019 and reaching 22,714 citations in 2020. Despite a mild decrease in the annual publications in 2019, the rate of increase in citations remained dramatically high, indicating the high quality of the publications on cancer gene therapy.

Disciplines involved in cancer gene therapy were detected by co-occurrence analysis of disciplinary categories through CiteSpace. It was shown in the pie chart (Figure 2B) that Oncology (10.4%), Research and Experimental Medicine (7.6%), and Pharmacology and Pharmacy (6.5%) were the top three most popular categories during 2016–2020. Furthermore, according to the centrality, the top three subjects were Biotechnology and Applied Microbiology (centrality = 0.31), Biochemistry and Molecular Biology (centrality = 0.28) and Oncology (centrality = 0.20), indicating that these subjects held a dominant place in the field of cancer gene therapy during 2016–2020. Compared with the distribution of disciplines during 2011–2015, which is presented in Figure 2C, it is noteworthy that in the recent 5 years, there was a huge increase in the account of publications on Materials Science. Moreover, Nanoscience and Nanotechnology first entered the top 10 during 2016–2020 with a 3.5 percent account. It is evident that gene therapy for cancer is a multidisciplinary field covering a wide range of interests, such as oncology, pharmacology, nanotechnology, materials science, etc.

### Analysis of Authors and Cited Authors

**Figure 3:**
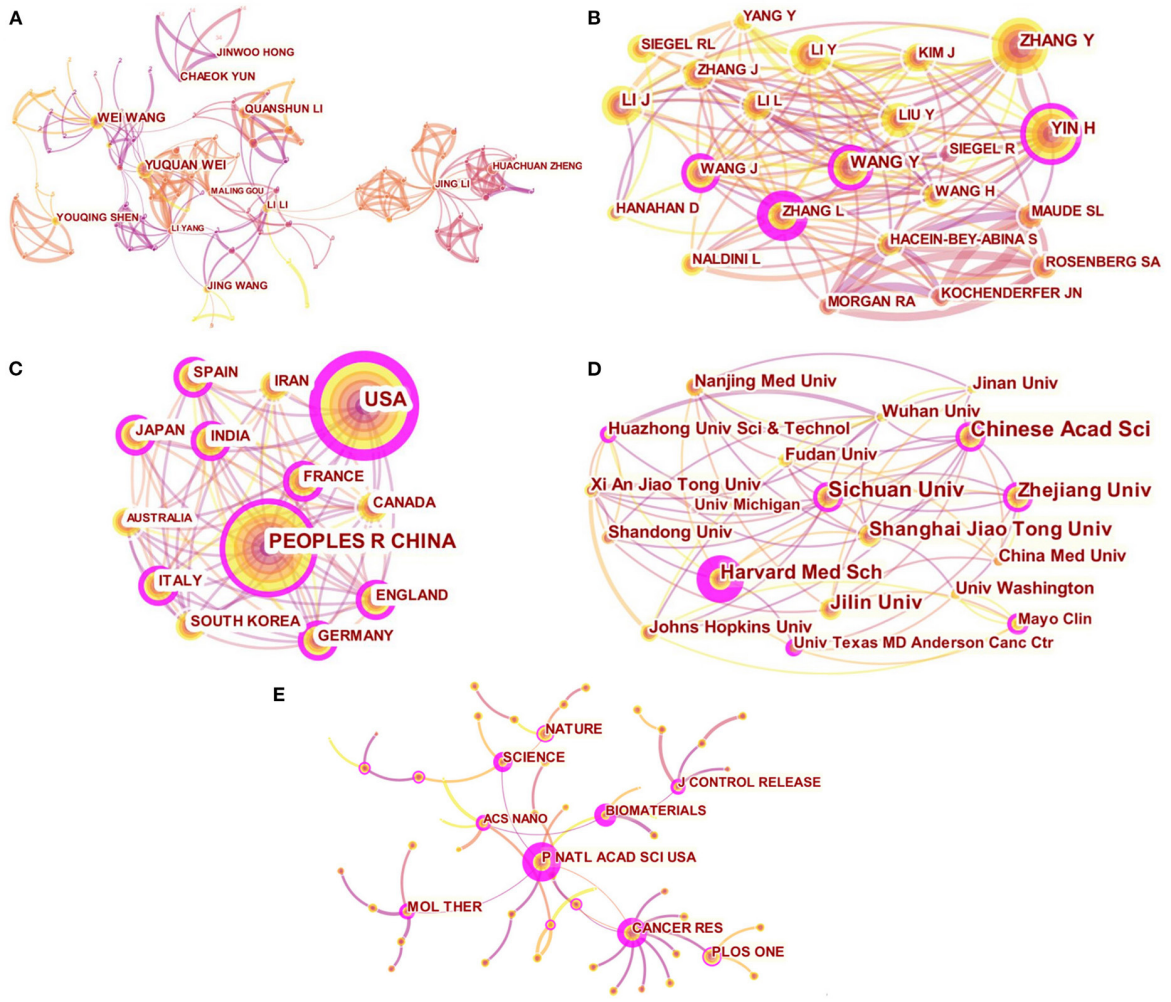
Distribution of the high-impact authors, institutions, countries and journals related to cancer gene therapy. **(A)** Network map of authors; **(B)** Network map of co-cited authors; **(C)** Network map of countries; **(D)** Network map of institutions; **(E)** Network map of co-cited journals.

The top 10 authors and co-cited authors were listed in Table 1. WANG W published the highest number of papers (*n* = 24), followed by WEI Y (*n* = 23), YUN C (*n* = 17), and LI Q (*n* = 15). Li L (0.12) and Yang L (0.11) were the authors with a centrality of more than 0.1. The core researchers as team leaders and their cooperation relationship were shown in the network map (Figure 3A). In terms of co-citation times (Table 1), ZHANG Y (*n* = 308) ranked first, followed by YIN H (*n* = 304), LI J (*n* = 282), and WANG Y (*n* = 250). The relationship of the co-cited authors was presented in Figure 3B. YIN H (centrality = 0.19), ZHANG L (centrality = 0.16) and WANG X (centrality = 0.16) were the top 3 authors according to the centrality, indicating their significant impact on the cancer gene therapy research.

**Table 1:** The top 10 authors and cited authors contributed to cancer gene therapy.

**No**.	**Author**	**Publications**	**Cited author**	**Citation times**
1	Wang Y	24	Zhang Y	308
2	Wei Y	23	Yin H	304
3	Yun C	17	Li J	282
4	Li Q	15	Wang Y	250
5	Hong J	14	Li Y	241
6	Wang J	13	Liu Y	217
7	Shen Y	13	Wang J	203
8	Xu F	13	Siegel Rl	192
9	Zheng H	12	Kim J	189
10	Li L	11	Naldini L	185

### Analysis of Countries and Institutions

A total of 58 countries were found to contribute to cancer gene therapy, of which the top 10 countries combined account for 75.4% of all articles published. The top 10 countries and institutions on cancer gene therapy were listed in Table 2. The top three countries with the most publications were PEOPLES R CHINA (*n* = 1,693), USA (*n* = 1,203), and GERMANY (*n* = 215). The top three countries in centrality were the USA (centrality = 0.30), ITALY (centrality = 0.17) and PEOPLES R CHINA (centrality = 0.16), indicating the high impact of these three countries in this field. The collaboration between the countries was shown in Figure 3C.

**Table 2:** The top 10 countries and institutions contributed to cancer gene therapy.

**No**.	**Country**	**Publications**	**Institution**	**Publications**
1	Peoples R China	1,693	Sichuan Univ	107
2	USA	1,203	Chinese Acad Sci	101
3	Germany	215	Shanghai Jiao Tong Univ	88
4	South Korea	197	Zhejiang Univ	82
5	Japan	190	Jilin Univ	82
6	Iran	183	Harvard Med Sch	78
7	England	178	Sun Yat Sen Univ	65
8	Spain	156	Tabriz Univ Med Sci	52
9	India	141	Johns Hopkins Univ	51
10	Italy	138	Nanjing Med Univ	47

Table 2 presented that Sichuan University published the highest number of articles (*n* = 107), followed by Chinese Academy of Sciences (*n* = 101), Shanghai Jiao Tong University (*n* = 88), and Zhejiang University (*n* = 82). In terms of centrality, Harvard Medical School ranked first with 0.48. Other institutions with a centrality of more than 0.1 included Sichuan University (centrality = 0.12), Mayo Clinic (centrality = 0.12), University of Texas MD Anderson Cancer Center (centrality = 0.12), and Chinese Academy of Sciences (centrality = 0.11), highlighting the dominant place of these institutions in the field of cancer gene therapy. The collaborative network among the institutions showed the research on cancer gene therapy in the United States and China were led by the Harvard Medical School and Sichuan University, respectively (Figure 3D).

### Analysis of Journals and Cited Journals

Table 3 listed the top 10 productive journals from 2016 to 2020, which published a total of 656 papers. *Oncotarget* was the journal publishing the largest number of literature (*n* = 99), followed by *J Control Release* (*n* = 74), *Oncol Lett* (*n* = 74), and *Hum Gene Ther* (*n* = 70). Additionally, the co-citation analysis of journals revealed that *Cancer Res* had the highest co-citation times (*n* = 2,405), followed by *P Natl Acad Sci USA* (*n* = 2,397), *Nature* (*n* = 2,078), and *PLoS ONE* (*n* = 2,073). The journals with a centrality ≥0.1 were *P Natl Acad Sci USA* (centrality = 0.52), *J Control Release* (centrality = 0.33), *Biomaterials* (centrality = 0.22), *Cancer Res* (centrality = 0.18), and *Gene Ther* (centrality = 0.13). The journal analysis visually showed *P Natl Acad Sci USA* as the core journal in the field of cancer gene therapy (Figure 3E).

**Table 3:** The top 10 journals and cited journals contributed to cancer gene therapy.

**No**.	**Journal**	**Publications**	**Cited journal**	**Citation times**
1	*Oncotarget*	99	*Cancer Res*	2,405
2	*J Control Release*	74	*P Natl Acad Sci USA*	2,397
3	*Oncol Lett*	74	*Nature*	2,078
4	*Hum Gene Ther*	70	*PLoS ONE*	2,073
5	*Cancer Gene Ther*	63	*Mol Ther*	1,891
6	*Int J Mol Sci*	61	*Science*	1,856
7	*Sci Rep*	61	*Clin Cancer Res*	1,715
8	*Int J Nanomedicine*	56	*Gene Ther*	1,622
9	*Biomaterials*	49	*J Control Release*	1,521
10	*Cancers (Basel)*	49	*Cell*	1,514

### Analysis of Keywords

**Figure 4:**
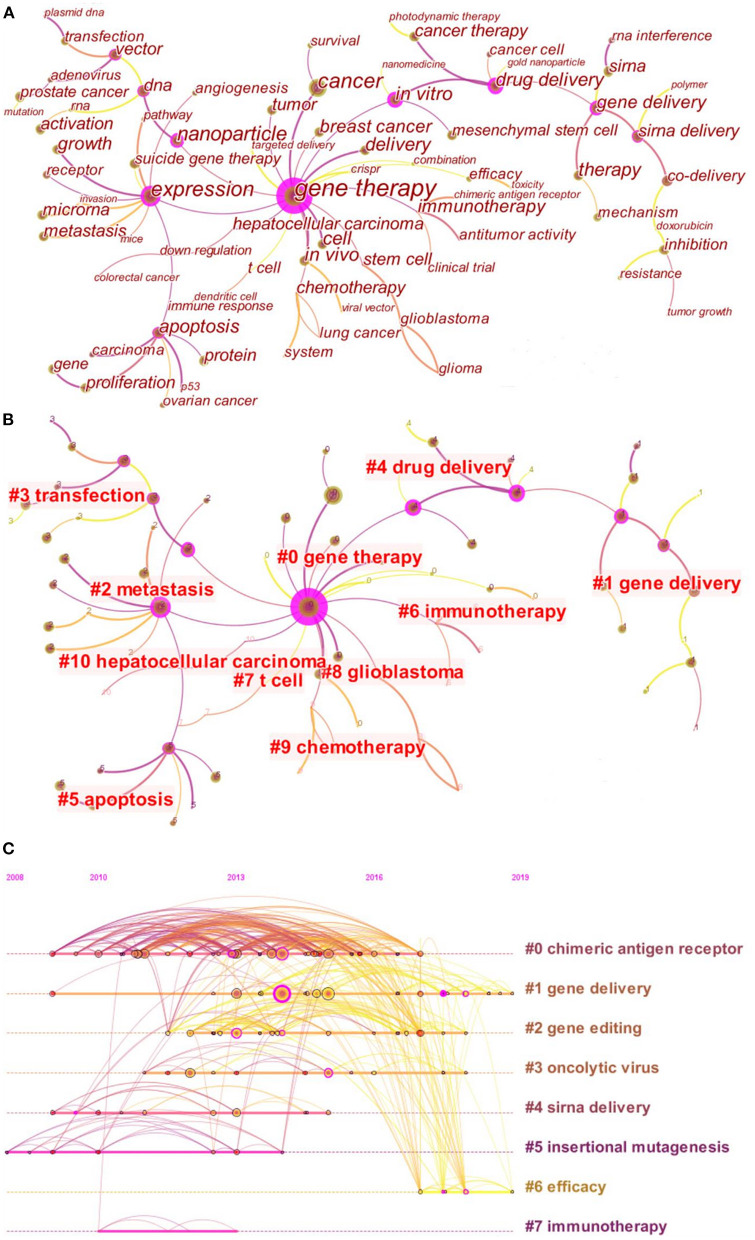
Analysis of keywords and co-cited references. **(A)** Co-occurrence network map of keywords; **(B)** Cluster map of keywords. Each nodes represented a keyword. All the keywords were categorized into 11 clusters, among which the cluster#0, #1, and #4 were the latest (mean year = 2017). The cluster label was the central keyword among all keywords included in the cluster; **(C)** Timeline view of co-cited references. Each node represented a cited reference, and the number of citation was reflected by the node size. The node volumes of the cluster decreased gradually from cluster#0 to cluster#6. The label of each cluster represented the main topic of the references included in the cluster. The clusters could be ranked by mean year from oldest to latest, namely #5 (2010), #7 (2011), #4 (2012), #0 (2013), #2 (2014), #3 (2014), #1 (2016), and #6 (2018).

The co-occurrence analysis of keywords presented the keywords with high frequency to reveal the main topic in the field of cancer gene therapy (Figure 4A). The “gene therapy” was the most frequent keyword and held the central position in the network map. In the clustering analysis, the co-occurrence keywords were into 11 clusters (Figure 4B). The three clusters labeled “gene delivery,” “gene therapy,” and “drug delivery” were the latest (mean year = 2017), indicating the frontiers of research in the field of cancer gene therapy. Furthermore, the burst keywords were detected, which referred to keywords with a sharp increase in frequency. The keywords with the strongest citation bursts were listed in Table 4. The keyword “pathway” had the highest burst strength, followed by “down regulation,” “clinical trial,” “angiogenesis,” and “lung cancer.”

**Table 4:** The keywords with the strongest burst on cancer gene therapy.

**Keywords**	**Strength**	**Begin**	**End**
Angiogenesis	12.2	2016	2017
Lung cancer	10.06	2016	2017
Adenovirus	6.29	2016	2017
Down regulation	15.01	2017	2018
Clinical trial	13.87	2017	2018
Cancer cell	6.33	2016	2018
Antitumor activity	5.87	2016	2018
Rna interference	5.23	2016	2018
Glioma	4.26	2016	2017
Pathway	16.04	2018	2020
Ovarian cancer	7.5	2017	2020

### Analysis of References

Co-cited references are references that have been co-cited in a set of publications. By clustering, all the co-cited references were divided into eight clusters, which were presented in form of a timeline view (Figure 4C). The latest cluster was cluster#6 (mean year = 2018), followed by cluster#1 (mean year = 2016). The latest cluster was labeled “efficacy,” indicating that the cited references of cluster#6 earned excessive attention in recent years. In cluster#6, there were six highly-cited references published during 2017–2019, of which the detailed information was listed in Table 5. Furthermore, the top 10 co-cited references with the strongest burst were listed in Table 6. Among these co-cited references, “Siegel (2014)” had the strongest strength of burst, followed by “Whitehead (2009),” “Mintzer (2009),” and “Johnson (2009).”

**Table 5:** The cited references of the cluster #6 in the reference analysis.

**No**.	**Title**	**Journal**	**First author**	**Year**
1	Tisagenlecleucel in Children and Young Adults with B-Cell Lymphoblastic Leukemia	*New Engl J Med*	Maude SL	2018
2	Axicabtagene Ciloleucel CAR T-Cell Therapy in Refractory Large B-Cell Lymphoma	*New Engl J Med*	Neelapu SS	2017
3	Long-Term Follow-up of CD19 CAR Therapy in Acute Lymphoblastic Leukemia	*New Engl J Med*	Park JH	2018
4	CAR T cell immunotherapy for human cancer	*Science*	June CH	2018
5	Tisagenlecleucel in Adult Relapsed or Refractory Diffuse Large B-Cell Lymphoma	*New Engl J Med*	Schuster SJ	2019
6	Chimeric Antigen Receptor Therapy	*New Engl J Med*	June CH	2018

**Table 6:** The top 10 co-cited references with the strongest burst on cancer gene therapy.

**No**.	**Title**	**Strength**	**Journal**	**First author**	**Year**
1	Cancer statistics, 2014	10.81	*Ca-Cancer J Clin*	Siegel R	2014
2	Knocking down barriers: advances in siRNA delivery	10.68	*Nat Rev Drug Discov*	Whitehead KA	2009
3	Nonviral vectors for gene delivery	10.68	*Chem Rev*	Mintzer MA	2009
4	Gene therapy with human and mouse T-cell receptors mediates cancer regression and targets normal tissues expressing cognate antigen	10.68	*Blood*	Johnson LA	2009
5	B-cell depletion and remissions of malignancy along with cytokine-associated toxicity in a clinical trial of anti-CD19 chimeric-antigen-receptor-transduced T cells	8.98	*Blood*	Kochenderfer JN	2012
6	Transfusion independence and HMGA2 activation after gene therapy of human β-thalassaemia	8.41	*Nature*	Cavazzana-Calvo M	2010
7	Cancer statistics, 2013	8.13	*Ca-Cancer J Clin*	Siegel R	2013
8	Evidence of RNAi in humans from systemically administered siRNA *via* targeted nanoparticles	8.13	*Nature*	Davis ME	2010
9	Hematopoietic stem cell gene therapy with a lentiviral vector in X-linked adrenoleukodystrophy	7.28	*Science*	Cartier N	2009
10	Randomized dose-finding clinical trial of oncolytic immunotherapeutic vaccinia JX-594 in liver cancer	7	*Nat Med*	Heo J	2013

## Discussion

In the present study, we utilized CiteSpace to conduct a bibliometric analysis of the global scientific outputs on cancer gene therapy published during 2016–2020. The records obtained from the Web of Science Core Collection were analyzed from multiple perspectives and the results were presented in the tables and knowledge network maps. The results showed that the academic attention from multiple subjects on cancer gene therapy grew at a dramatic speed from 2016 to 2020 according to the annual citations and analysis of the discipline distribution. Notably, Materials Science and Nanoscience and Nanotechnology took an increasing part in the research on cancer gene therapy, which may become the promising impetus for the development of this field. Additionally, WANG W was the most productive author, while ZHANG Y ranked top in terms of citations. The USA and PEOPLES R CHINA were the top two countries contributing to cancer gene therapy. Correspondingly, a majority of active institutions were from these two countries, among which Harvard Med Sch and Sichuan Univ ranked top. The detection of burst keywords suggested that “ovarian cancer” might be the potential direction for future research.

Of note, the top three latest clusters were labeled “gene delivery,” “drug delivery,” and “gene therapy” in the clustering analysis of keywords. We also noticed that in the reference analysis, the cluster#2 labeled “gene delivery” was dominant in terms of both node volume and mean year. Considering these results together, the keyword “gene delivery” was thought to best reflect the research frontier on cancer gene therapy. Besides, the detection of burst keywords showed that “ovarian cancer” was the latest burst keyword, indicating the potential research directions. Hence, we conducted a brief literature review on the top five most cited papers that cited “gene delivery” or “ovarian cancer” as the keyword.

### Gene Delivery

In recent years, multiple non-viral vectors have been proposed to achieve efficient delivery of therapeutic genes to targeted tissues and cells. Novel delivery systems based on nanomaterials have gained special concern for their advantages over viral vectors, such as less immune response, low cytotoxicity, designing flexibility, etc., (26). Tatiparti et al. suggested that nanoparticle was the most common approach to deliver siRNA to the targeted tumor cells, which served to suppress the oncogenes by gene silencing mechanism (27). Besides, Riley et al. classified the nanomaterials into five types by their nature, including inorganic, graphene, proteins and peptides, lipids and polymer, of which the characteristics were then introduced (26). In the review by Zhou et al., the vector surface, size and stability were summarized as three dilemmas for nanoproperties, which largely affected the transfection efficiency during the five-step cascade of gene delivery (28). Although there were several strategies, such as shielding functional groups, charge-reversal surface, etc., to modify the nanoproperties, the overall gene expression efficiency of nanomaterials-based vectors remained far lower than that of viral vectors (28). The research topics in these highly-cited publications reflect the significant impact of Materials Science and Nanoscience and Nanotechnology on the development of cancer gene therapy, which is consistent with the results of the disciplinary distribution analysis. On the other hand, compared with viral vectors, higher transfection efficiency could be achieved by electroporation, which was a promising technique for both gene therapy and DNA vaccines (29). Recently, Liang et al. succeeded in delivering functional miR-26a to liver cancer cells by engineered exosomes, indicating the potential role of exosomes to deliver therapeutic miRNA species to tumor cells (30).

### Ovarian Cancer

With the rapid progress of gene therapy for ovarian cancer, various strategies have been feasible technically and tested by a number of clinical trials in recent years (31). The review by Ayen et al. comprehensively compared the targeted genes, advantages and weaknesses of seven gene therapy strategies for ovarian cancer, including tumor suppressor gene therapy, oncofactor inhibition strategy, suicide gene therapy, antiangiogenic gene therapy, genetic immunopotentiation, multi-drug resistance and oncolytic virotherapy (31). Additionally, it was indicated in the review that further investigation on the gene therapy targeting the biomarkers of ovarian cancer stem cells was in need since it was considered one of the effective approaches to reduce drug resistance and relapse rate (31). In another review, Worku et al. summarized the role of long non-coding RNAs in the progress of ovarian cancer and considered *HOST2* and *let-7b* as potential therapeutic targets (32). It was suggested that the regulation of some long non-coding RNAs, such as *HOTAIR, UCA1, HOXA11AS*, etc., through RNAi techniques or CRISPR/Cas-9 was thought to be a promising therapy for ovarian cancer (32). He et al. also demonstrated that CRISPR-Cas9 targeted *DNMT1* had the potential to be a therapeutic regimen for ovarian cancer (33). Besides, Vandghanooni et al. delivered cisplatin and anti-miR-21 to targeted ovarian cancer cells by nanoparticles, which led to the inhibition of miR-21 and increased effects of chemotherapeutics, providing a potential combination therapy for ovarian cancer (34). On the other hand, ovarian cancer is regarded as an immunogenic tumor with neoantigens that are not present in normal tissues. Recently, Liu et al. identified two mutated genes, *NUP214* and *JAK1*, as biomarkers for recognition of ovarian cancers by genomics and bioinformatics approaches and introduced a novel strategy to screen the neoantigens in the ovarian cancer tissue, with a validation rate of 19%, which was far higher than that of the traditional method (35).

Admittedly, there were some limitations in the present study, such as a lack of literature from non-English databases and the potential bias caused by self-citation. Nonetheless, our study presented the current status and frontiers of research on cancer gene therapy from 2016 to 2020 and provided potential directions for future research. Therefore, the relevant scholars, clinicians and students would greatly benefit from the bibliometric analysis in this paper.

## Data Availability Statement

The original contributions presented in the study are included in the article/supplementary material, further inquiries can be directed to the corresponding author/s.

## Author Contributions

YLi conceived and designed the structure of this manuscript and revised the paper. SH, AA, YLai, HH, and ZC wrote the paper. All authors contributed to the article and approved the submitted version.

## Funding

This study was supported by National Natural Science Foundation of China (NSFC) Grant No. 81972546.

## Conflict of Interest

The authors declare that the research was conducted in the absence of any commercial or financial relationships that could be construed as a potential conflict of interest.

## Publisher's Note

All claims expressed in this article are solely those of the authors and do not necessarily represent those of their affiliated organizations, or those of the publisher, the editors and the reviewers. Any product that may be evaluated in this article, or claim that may be made by its manufacturer, is not guaranteed or endorsed by the publisher.
